# Physical self-concept and ability to swim in patients born with anorectal malformation and Hirschsprung’s disease: a case control study

**DOI:** 10.1186/s12887-022-03782-5

**Published:** 2022-12-15

**Authors:** Tatjana Tamara König, Mattis Krude, Oliver J. Muensterer

**Affiliations:** 1 Department of Pediatric Surgery Universitätsmedizin, Johannes Gutenberg-University Mainz, Langenbeckstr. 1, 55131 Mainz, Germany; 2 Department of Pediatric Surgery, Ludwig-Maximilians-University Munich, Dr. Von Haunersches Kinderspital, Munich, Germany

**Keywords:** Anorectal malformation, Hirschsprung’s disease, Physical self-concept, Swimming, Physical activity

## Abstract

**Background:**

Children with anorectal malformation (ARM) and Hirschsprung’s Disease (HD) live with permanent urinary and bowel symptoms, possibly impairing motor development in early childhood. Not being able to swim adds an unnecessary health risk. The aim of this study was to determine the ability to swim and physical self-concept in patients with ARM and HD.

**Methods:**

We performed an anonymous survey among the members of the national patient organization SoMA e.V. (6 through 25 years). A control group was recruited from our department. Ability to swim, symptom load according to Rintala Score and physical self-concept were recorded using validated questionnaires. Patients were matched with controls according to gender and age. Mean scores and 95%-confidence intervals (95%-CI) were calculated, χ^2^-test and multiple linear regression models were used as appropriate.

**Results:**

Totally, 83 match-control-pairs were included. Patients learned to swim at a similar age and rate (6.5 years, 95%-CI: 6.1–6.9, 74.7% swimmers) compared to controls (6.4 years, 95%-CI: 6.1–6.8, 79.5% swimmers, *p* = 0.46). VACTERL patients had a significantly lower swimmer rate (59.1%, *p* = 0.048). Swimmers had a significantly higher mean Rintala Score (12.5, 95%-CI: 11.6–13.2) compared to non-swimmers (10.4, 95%-CI: 8.1–12.1, *p* = 0,049). In prepubertal children (6 through 12 years), no difference in physical self-concept was shown compared to controls. Adolescents and young adults with ARM/HD, especially females, had a significantly lower mean score for the subscales of flexibility, speed, endurance and sports competence, independent of bowel symptom load according to Rintala Score.

**Conclusions:**

Patients with ARM/HD have normal swimming skills and a normal physical self-concept in childhood that decreases with age compared to peers. In adolescence, parents and health care professionals should actively promote physical activity in ARM/HD patients.

**Supplementary Information:**

The online version contains supplementary material available at 10.1186/s12887-022-03782-5.

## Background

In spite of ongoing medical and surgical advances, children born with anorectal malformation (ARM) or Hirschsprung’s disease (HD) often require life-long bowel management for fecal and urinary incontinence, as well as constipation. The Rintala Score is an established tool to objectify severity of bowel symptoms and outcome [1].

The term „anorectal malformation “ includes a wide spectrum of anatomic presentations [2]. Longterm health-related quality of life (HrQOL) and fecal continence in patients born with ARM are strongly depended on the type of malformation, concomitant sacral anomalies, development of the pelvic floor [1, 2], anorectal sensation, bowel motility and precise surgical reconstruction [2]. Soiling, mostly associated with constipation, is present in approximately 37% of patients [3], the prevalence of chronic constipation ranging from 22 to 87 percent [4], while fecal incontinence is present in 17 to 77 percent of patients born with ARM [4]. Furthermore, ARM is associated with other malformations, such as congenital heart disease, skeletal anomalies, and VACTERL association (vertebral defects, anal atresia, cardiac defects, tracheoesophageal fistula, renal anomalies, and limb abnormalities [2]), which may have a direct impact on physical activity and fitness on their own.

In patients with HD, there are usually no associated malformations. There are several surgical approaches to treat HD, all yielding similar long-term results [5]. According to a recent meta-analysis, the prevalence of fecal incontinence and constipation in patients older than ten years is 20 and 14 percent [5], respectively. Other studies report constipation in a quarter of patients [6]. While incontinence improves with age, symptoms of constipation and soiling seem to remain stable throughout adult age [7].

Frequent inpatient-treatments, presence of an ostomy or abdominal scarring, fecal and urinary incontinence, soiling and constipation may impact negatively on physical activity and sports participation, especially swimming. According to the World Health Organisation, physical inactivity has become one of the leading causes of death worldwide [8]. Not being able to swim poses an additional substantial health hazard, especially in children with chronic disease. There is no standardized definition of „being able to swim “, compromising comparability of studies [9]. In one study on 9750 representative German children and adolescents aged five through 17 years, 14.5 percent of participants were not able to swim. The average age for gaining swimming proficiency was six years [9]. Girls were more likely to be able to swim than boys at primary school age, and on average learned to swim 4 months earlier [9]. Low socio-economic status (odds-ratio 5.95) and a background of migration (odds-ratio 2.39) were identified as risk factors for a lack of swimming skills [9].

The physical self-concept consists of subjective physical attractiveness and perceived physical abilities [10]. It is considered indicative for the level and maintenance of physical activity, general physical fitness and development of obesity and correlates with life-satisfaction levels [11].

The aim of this study is to determine the impact of being born with ARM or Hirschsprung’s Disease on the ability to swim and physical self-concept among patients, taking into account clinical symptoms and incontinence scores.

## Methods

### Study design

From November 2019 to May 2020, an anonymous online-survey was conducted among the members of the German national ARM and HD patients support group SoMA. e.V. (www.soma-ev.de). A total of 280 members aged 6 to 25 years were invited to participate via e-mail newsletter issued by SOMA e.V. Simultaneously, a control group of children and adolescents was recruited at the Department of Pediatric Surgery at the Medical Center of the Johannes Gutenberg-University Mainz by publicly accessible posters in the out-patients clinic and on the ward. Patients with congenital malformation or bowel disease were excluded. Patients and controls were matched according to gender and age. All participants older than 18 years were defined as adults and matched accordingly.

Participants who reported being able to swim at least 25 m without pause were defined as “swimmers”. Basic swimming skills were defined on the basis of the German swimming badge (“*Seepferdchen*”, Bronze swimming badge), advanced swimming skills were defined as having passed the requirements for an advanced German swimming badge (Silver, Gold, Life-Guard). Questionnaires lacking crucial information, such as gender, age, diagnosis, data regarding the ability to swim, or physical self-concept were excluded.

### Survey instruments

The questionnaire for ARM/ HD patients included items regarding patient history, current health status including fecal incontinence and constipation according to the Rintala Scale [1], and a standardized validated questionnaire to test physical self-concept. The Rintala Scale includes 7 items (ability to hold back defecation, feeling urge to defecate, frequency of defecation, soiling, accidents, constipation and social problems). The score ranges between 1 and 20 points, 20 being the most favorable outcome. In patients with ARM, the presence of additional vertebral, cardial, tracheoesophageal or limb malformation was queried separately. Presence of two or more of these malformations in addition to the ARM were defined as VACTERL association.

The physical self-concept was assessed using age-appropriate validated questionnaires (*Physisches Selbst-Konzept,* [PSK] [10] for patients age 13–25 and *Physisches Selbstkonzept—Kinder* [PSK-K] for patients aged 5–12 years [11]). Both the PSK and the PSK-K consist of seven subscales measuring subjective physical abilities (Table 1), and physical appearance [10, 11], each phrased appropriately for the targeted age and measured on a bipolar 4-point Likert scale. For analysis, negatively worded items were reversely scored. The questionnaire for the control group included a general medical history instead of disease-specific questions. The items regarding the ability to swim and the physical self-concept (PSK or PSK-K according to age) were identical to the patient group.

**Table 1:** Subscales and composition of the PSK-K [10] and PSK [11]

Sub-Scales PSK-K/ PSK	exemple	n items PSK-K	n items PSK
Physical appearence	"…like to look at myself in the mirror…"	3	10
Sports competence	"…am good at sports…"	3	6
Endurance	"…can move for a long time withou getting tired…"	3	6
Speed	"…run faster than others…"	3	6
Strength	"…am strong"	3	6
Coordination	"…easily control my bodies movements…"	3	6
Flexibility	"…can bend in all directions…"	3	6

### Statistical analysis

For continuous variables, means and 95%-confidence intervals (95%-CI) were calculated. In case of non-overlapping 95%-CI, the difference was considered statistically significant. For ordinal data, χ^2^-Test was used. *p* < 0.05 was considered statistically significant. Furthermore, multiple linear regression models were used, as appropriate. In order to evaluate the physical self-concept for all ages, percentage of maximum score was calculated for PSK and PSK-K scores (PSK%) and for the subscale of physical appearance (PA%).

## Results

### General

A total 83 match-control pairs were included in the analysis (return rate patient group 29.6%, female *n* = 32, male *n* = 51). In the patient group, two thirds (69.9%) of questionnaires were answered by a proxy, 18.1% by the patient and 10.8% by a patient with the help of a proxy. None of the PSK-K results were reported by the patients alone (85% by proxy, 15% by proxy and patient), while approximately half of PSK results (48%) were self-reported.

In the control group, 47.0% of questionnaires were answered by a proxy alone, while 38.6% were answered by the participant and 12.0% were answered by proxy and participant together. In children below the age of 13 (PSK-K group), 19.1% were self-reported, 63.8% answered by a proxy and 14.9% answered by a participant with help of a proxy. In adolescents and young adults, the vast majority (63.9%) was self-reported. In the control group, five children had asthma, two atopic dermatitis, one attention deficit hyperactivity disorder and epilepsy and one suffered from scoliosis, one from migraines and one from depression.

### Patient history

In the patient group, 18 patients were born with HD and 65 with ARM, including 22 patients with VACTERL association. In most HD of the 18 patients, the disease extended to the rectosigmoid and distal colon (*n* = 10). In the remaining patients, rectum (*n* = 1), rectosigmoid (*n* = 2) were affected, in addition to total colonic aganglionosis in one patient. In 4 patients, the extend of the disease was not specified by the participant. Females with ARM had cloaca (*n* = 9) or cloacal extrophy (*n* = 7), vaginal or vestibular fistula (*n* = 8) or perineal fistula (*n* = 6). Most males were born with rectourethral or rectoprostatic fistula (*n* = 17), rectovesical fistula (*n* = 4), perineal fistula (*n* = 7), or cloacal extrophy (*n* = 3). Tree patients had genetic syndromes. One patient with HD had Down syndrome. In the ARM group, there was one patient with Cat-Eye syndrome and one with Bardet-Biedl syndrome. Two thirds (68.7%) had more than three procedures with general anesthesia for treatment of ARM/HD. The vast majority of patients (*n* = 68) had had an ostomy in infancy, five patients still had an ostomy. The mean Rintala Score was 11.9 (95%-CI: 11.1–12.7). There was no significant difference between children (5–12 years, 11.6, 95%-CI: 10.6–12.5) and adolescents/ young adults (13 through-25 years, 12.4, 95%-CI: 11.1–13.7).

### Ability to swim

The vast majority of participants enjoyed swimming (Table 2). Fewer children in the patient group were swimmers (74.7%) compared to controls (79.5%, χ^2^ *p* = 0.46). In the subgroup analysis, the difference was only statistically significant for patients born with VACTERL association (40.9% not able to swim, χ^2^ *p* = 0.048). Reasons for disliking swimming in the patient group were fear of water (*n* = 2), hesitancy to show their body (*n* = 3) or general dislike (*n* = 7). Only one patient reported an “accident” regarding fecal incontinence while swimming. Patients learned to swim at a similar age compared to controls (Table 2) and were more likely to have participated in a learn-to-swim-course than controls (Table 2). In the patient group, significantly more parents had a higher education, while in the control group significantly more parents had migration background (Table 2).

**Table 2:** Swimming skills and general demographics of study participants (^*^significant difference, ^**^adult patients older than 18 years were matched with other adults)

	**Patients**	**Controls**	**p**
male (n)	51		
female (n)	32		
Mean age (years) (standard deviation, minimum–maximum)^**^	13.4 (5.9, 6–25)	12.8 (5.0, 6–24)	
Mean age PSK-K (years) (standard deviation, minimum–maximum)	8.9 (2.0, 6–12)	8.9 (2.0, 6–12)	
Mean age PSK (years) (standard deviation, minimum–maximum)^**^	19.3 (3.5, 13–25)	17.9 (2.7, 13–24)	
**Swimming skills**			
enjoy swimming	89.2%	91.6%	0.60
participated in learn-to-swim course	78.3%	62.7%	0.03^*^
mean age being able to swim (95%-CI)	6.5 (6.1–6.9)	6.4 (6.1–6.8)	
non-swimmers	25.3%	20.5%	0.46
basic swimming skills	54.2%	50.6%	0.88
advanced swimming skills	20.5%	28.9%	0.21
**Higher education parents**			
no	55.4%	67.5%	< 0.05^*^
one parent	31.3%	24.1%	
both parents	13.3%	8.4%	
**Migration background**			
patient	6.0%	3.6%	0.47
one parent	12.0%	6.0%	< 0.05^*^
both parents	6.0%	6.0%	

Patients who could swim had a significantly higher mean Rintala score (12.5, 95%-CI: 11.6–13.2, *n* = 62) compared to patients, who could not swim (10.4, 95%-CI: 8.1–12.1, *n* = 21, p = 0,049). There was no difference in the Rintala score between patients with basic (mean 12.5, 95%-CI: 11.4–13.5) and advanced (mean 12.1, 95%-CI: 10.6–13.5) swimming skills. Out of the 5 patients presently having an ostomy: three were swimmers, two were non-swimmers.

### Physical self-concept in children 6 to 12 years (PSK-K)

**Fig. 1:**
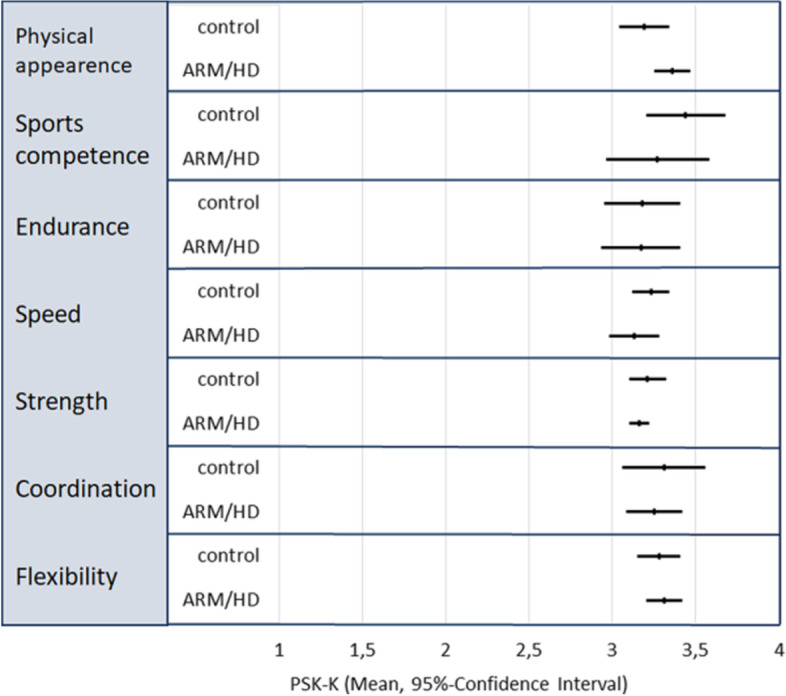
Mean physical self-concept score and 95%-confidence interval for separate subscales for physical self-concept in children 5 through 12 years in patients (ARM/HD) and controls (range 1 to 4 points). All values lie in the upper section of the scale. There were no significant differences between patients or controls. (PSK-K = physical self-concept-*Kinder*, ARM = anorectal malformation, HD = Hirschsprung’s disease)

**Fig. 2:**
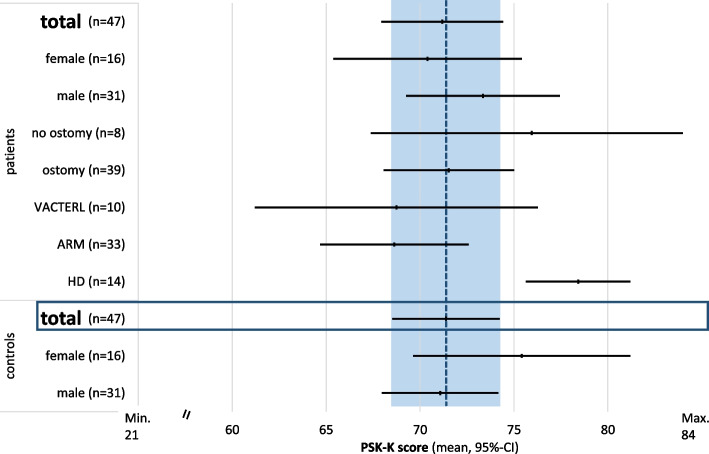
Mean physical self-concept total score and 95%-confidence interval in children 5 through 12 years in patients (ARM/HD) and controls (range 21 to 84 points). There were no significant differences between the mean (dotted line) and 95%-confidence interval (blue box) of the control group compared to patients, including influencing factors, such as gender or being able to swim. The PSK-K score of patients with HD was significantly higher compared to all other subgroups. (PSK-K = physical self-concept-*Kinder*, HD = Hirschsprung’s disease, ARM = anorectal malformation, VACTERL = VACTERL association)

A total of 47 case–control pairs were included in the PSK-K group according to age. The vast majority of questionnaires was reported by a proxy (*n* = 40) or proxy with patient (*n* = 7). There was no significant difference in mean PSK-K scores for any of the subscales (Fig. 1) or total PSK-K score (Fig. 2) between patients and controls. Over all, both patients and controls ranged in the upper section of the scale (Fig. 1). In the analysis for associated factors, there was no significant difference in total PSK-K score between genders, or patients with or without a prior ostomy (Fig. 2). Patients with HD had a higher PSK-K score compared to patients with VACTERL, ARM or even controls (Fig. 2).

### Physical self-concept in adolescents and young adults 13 to 25 years (PSK)

**Fig. 3:**
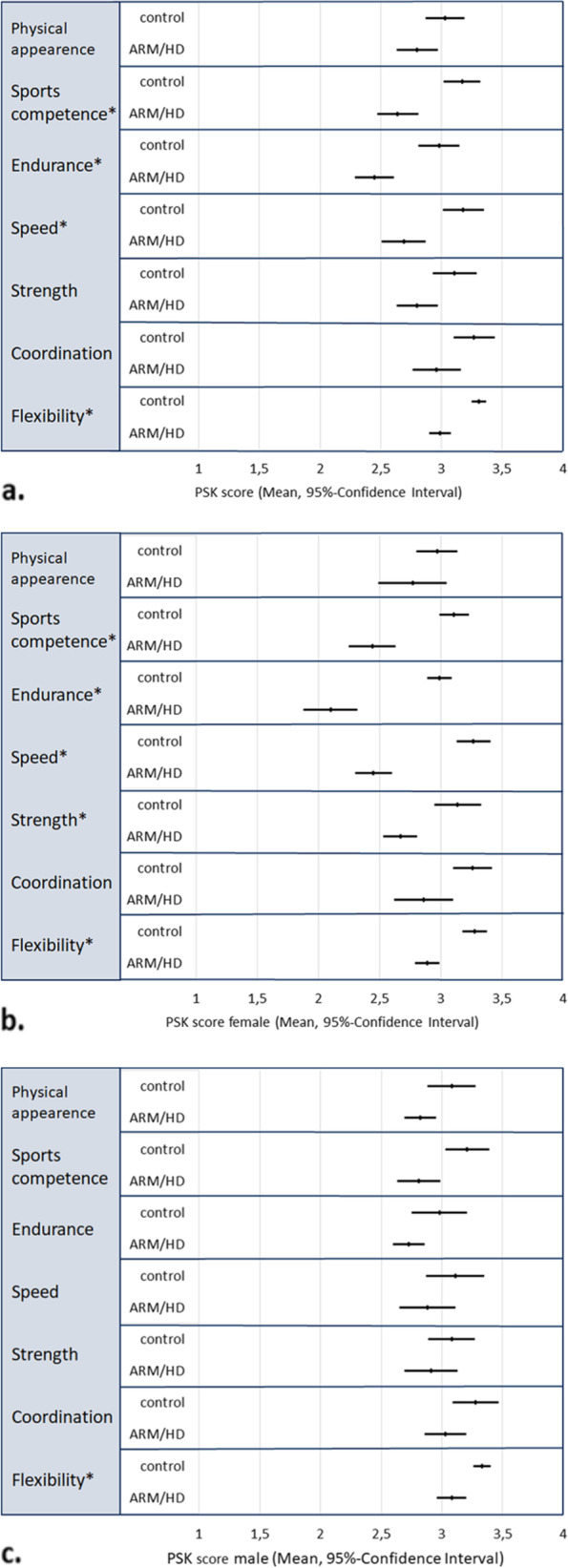
**a-c** Mean PSK score and 95%-confidence interval for separate subscales for physical self-concept in adolescents 13 through 25 years in patients (ARM/HD) and controls (range 1 to 4 points). **a**. In the analysis of both genders, the mean scores for sports competence, endurance, speed and flexibility were significantly lower compared to controls. **b**. In females, there was a significant and major deficit in the subscales for sports competence, endurance, speed, strength and flexibility. **c**. In males, only the score for flexibility was significantly reduced compared to controls. (* significant difference, PSK = physical self-concept, ARM = anorectal malformation, HD = Hirschsprung’s disease)

**Fig. 4:**
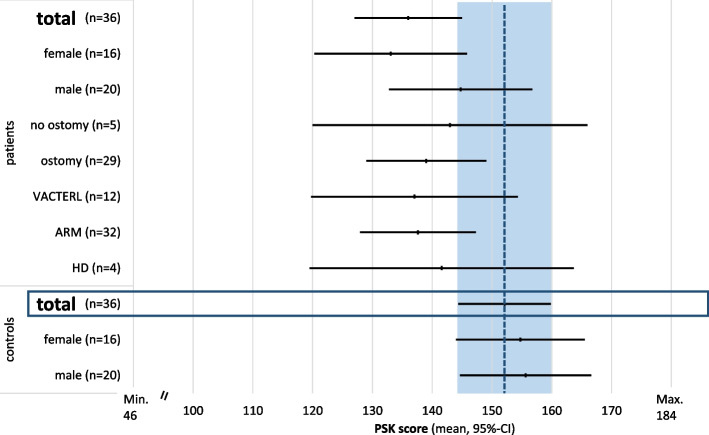
Mean physical self-concept total score and 95%-confidence interval in children 13 through 25 years in patients (ARM/HD) and controls (range 46 to 184 points). Even though a reduced physical self-concept score in patients compared to controls is evident, there were no significant differences between the mean (dotted line) and 95%-confidence interval (blue box) of the control group compared to patients, including influencing factors, such as gender or type of malformation. Patients, who are not able to swim have a significantly reduced physical self-concept score compared to controls. (PSK = physical self-concept, HD = Hirschsprung’s disease, ARM = anorectal malformation, VACTERL = VACTERL association)

In adolescents and young adults (*n* = 36 case–control pairs), controls had a higher mean PSK score in all subscales, compared to controls. There was a significant difference in mean scores for the subscales for flexibility, speed, endurance and sports competence. However, mean scores were still in the upper half of the scale (Fig. 3a). There was a greater difference in female than in male patients compared to controls of the same gender (Fig. 3b and c). In females, the differences in flexibility, strength, speed, endurance and sports competence were significant. For physical appearance, there was only a minor difference (Fig. 3b). In males, the only significant differences between patients and controls were seen for the subscales for flexibility and sports competence (Fig. 3c.). Not being able to swim in adolescence was associated with a lower mean PSK score, no other significant influencing factors could be identified (Fig. 4). In the control group, there was no difference in PSK scores between genders. Swimmers (ARM/HD and controls) had a significantly higher PSK score in all subscales, compared to participants, who could not swim.

### Symptom load and physical self-concept

In multiple linear regression, a statistically significant, but weak effect was shown for Rintala score or incontinence associated with ARM/ HD on PSK/ PSK-K score percentage (PSK%, adjusted r^2^ = 0.18, *p* < 0.01 respectively). For social problems, there was no significant correlation. The score percentage for the subscale of physical appearance (PA%) was analyzed separately. There also was a statistically significant, but weak effect on total Rintala score (*p* < 0.01), incontinence (*p* = 0.04, adjusted r^2^ = 0.06) without detectable effect of the social problems score.

Patients with VACTERL association hat a lower mean PSK% (68.8, 95%-CI: 61.9–75.4) compared to patients without VACTERL (78.3, 95%-CI: 74.8–81.9). However, PA% was comparable (VACTERL 76.4, 95%-CI: 70.1–82.8, no VACTERL 78.2, 95%-CI: 74.5–81.9). PA% was the same in children with prior ostomy (mean 76.8, 95%-CI: 72.9–80.7) compared to participants without ostomy in infancy (mean 74.6, 95%-CI: 65.5–83.6).

## Discussion

This is the first study to comprehensively address the ability to swim in ARM and Hirschsprung patients, along with physical self-concept using a standardized score. In this representative cohort both mild and severe cases of ARM and HD were included. Despite possible symptoms of incontinence, children with ARM or HD learned to swim at similar rates and ages compared to controls. Physical self-concept in prepubescent children was similarly high compared to healthy peers. Therefore, it comes as no surprise that younger children have no reservations against learning to swim at the same age as others. Only few patients reported bowel-related symptoms during swimming. However, swimmers had a significantly lower symptom load compared to non-swimmers. In the patient group, significantly more children attended learn-to-swim classes and there were significantly more parents with a higher education and fewer with migration background compared to the control group. Those factors may positively influence the probability of being able to swim in our cohort [9].

Cardiorespiratory fitness and muscular strength are positively correlated with the HRQoL in children and adolescents, including physical and psychological well-being and perceived health status [12]. A recent study evaluating sports performance revealed a significantly lower relative performance capacity in children with ARM in spiroergometry, independent of symptom load [13]. These results corroborate our own findings of subjectively reduced endurance in our patient cohort, independent of Rintala score.

Core somatic muscles are active before we are able to take a step [14]. Pelvic floor function is essential for core muscle integrity and influences pelvic and lumbar spine stability [14] and, therefore, posture, gait and pelvic tilt. Trunk load passes through the sacroiliac joint and is reinforced by transverse pelvic muscles [15]. In patients with pelvic floor and sacral anomalies, the entire system may be compromized. Pelvic floor dysfunction may promote urinary and fecal incontinence and pelvic pain [16] but may also impair sports competence. In female patients with ARM, defects in the pelvic floor muscles have been reported in all patients, even in lower forms of ARM [17]. A study assessing motor abilities of children aged 9 to 18 years reveal a significant deficit in all motor skills regarding the lower extremities and deep core muscles (lateral jump, long jump, crunches) [13]. In our patient cohort, adolescents had subjectively reduced motor function in terms of speed (males and females) and strength (females). While there is an abundance of studies on the impact of physical activity on pelvic floor function in postpartal or (post)menopausal women [16, 18–20], evidence for patients with ARM is scarce. Patients with HD had a better physical self-concept than even healthy children (Fig. 2). This finding is rather surprising and might be attributed by the fact, that HD patients have a normal pelvic floor in addition to bias caused by a small subsample size and possible selection bias. Further research on the influence of pelvic floor malfunction on locomotor performance in relation to deep core muscle and lower body strength is needed.

Children and especially adolescents with an ostomy face additional challenges, such as changes of body image and decreased independence that often affect the whole family [21]. After stoma-reversal, patients in our series had the same score for physical self-concept and subjective physical attractiveness as other patients and controls. Only five of the patients had a stoma at the time of the survey. For patients living with a stoma, a feasibility study on adults showed an increased HRQoL, stoma-related quality of life and self-efficacy after participation in a structured physical activity and health education program [22]. No stoma-related adverse events were reported [22]. While fecal and urinary incontinence are predictive of the HRQoL in patients with ARM/HD [23], physical self-concept and perceived physical attractiveness were largely independent of symptom load according to the Rintala score. This finding matches recent studies where lower scores for locomotor function and exercise capacity [13], as well as pelvic floor muscle defects [17] were observed without correlation to bowel symptom load. We conclude that physical activity may improve HrQOL in ARM/ HD patients independent of symptom load or presence of an ostomy.

Adolescence is considered as a crucial period in the development of the physical self-concept [24]. Overall, adolescents and young adults with ARM/HD had a lower score for physical self-concept compared to controls, which was significant for the subscales for flexibility, speed, endurance and sports competence, but notably, not physical appearance (Fig. 3a). In gender-specific analysis, female adolescents with ARM have a significantly lower physically related quality of life compared to males [25]. In regards to physical self-concept, healthy boys usually yield higher scores for the subscales for strength, endurance, speed and sports competence as children [11], as well as strength, speed and physical attractiveness as adolescents and young adults [10]. In gender-specific analysis of our cohort, male patients had significantly lower scores compared to male controls in only two areas (Fig. 3c), while females had significantly lower scores in multiple areas (Figs. 3b.) compared to female controls. Furthermore, female patients aged 13–25 years had significantly lower scores for strength, endurance and sports competence compared to male patients, while there was no gender difference in the control group. Interestingly, there was no significant difference in self-perceived physical appearance for neither male nor female patients nor controls in all age groups. Generally, more gender-specific surveys in regards to HrQOL and other subjective measures in patients with ARM/HD are needed in order to provide optimal personalized patient care.

In our study, patients with VACTERL association were a small, heterogeneous group that forbade further evaluation of subgroups. Cardial, vertebral and limb malformation may impair physical performance. However, it is safe to say, that these patients need special attention and physical activity must be promoted at an individual level to ensure safe participation in peer activities, including swimming.

The World Health Organization recommends a minimum of 60 min moderate to vigorous physical activity per day for all children and adolescents [26]. Children with ARM an HD start out with a normal physical self-concept, which changes around the onset of puberty, which is considered the crucial age for the development of physical self-concept [24]. Therefore, it is essential not to miss the point when physical activity in patients with ARM/ HD decreases compared to peers, especially in girls in order to initiate specialist care. Guidelines recommend a life-long follow-up even in asymptomatic patients with HD [27] and ARM [28]. However, many adolescents with ARM/ HD are lost to regular follow-up over time, particularly during the transition to adult care. Children and adolescents with chronic disease, especially females and patients with multiple health problems, e.g. VACTERL association, must be constantly encouraged to be physically active and learn to swim by families and health care workers to avoid adding further risk factors for their health.

## Limitations

Our study has some limitations. Firstly, there may be a certain selection bias. However, since this is true at the same rate for both groups, the comparison may still be considered valid. Secondly, the patient group was recruited from all of Germany, whereas the control group was recruited locally. Thirdly, many questionnaires were answered by a proxy alone. Imperfect agreement of proxy and patient reporting, especially for feelings or internalizing symptoms, is consistent in patients and healthy individuals [29]. While parents and caregivers might be aware of patients’ history and current symptoms, they may not know the true physical self-concept, especially in adolescents. Even though the children know their internal workings best, the information gained by both sides is equally valuable and must be considered [29]. A larger percentage of questionnaires in the patient group was answered by proxy alone compared to controls, which may lead to relevant bias. On the other hand, the fact that parents feel entitled to give this information about their children might indicate that patients might be less independent of their parents compared to healthy peers. In future studies, physical self-concept ought to be reported directly and in private by the affected subjects.

## Conclusion

Physical activity may have the potential to improve HrQOL, stoma-related quality of life and physical self-concept in children and adolescents with ARM or HD. Prepubescent children with ARM and HD have a normal physical self-concept according to caregivers and learn to swim at a normal age and rate largely independent of bowel-related symptom load. Only children with VACTERL association had a significantly increased risk of not being able to swim. Therefore, they should be screened for swimming ability routinely during follow-up. In adolescence, physical self-concept scores decrease compared to healthy peers, especially in female patients. These patients must be encouraged to stay physically active in order to avoid additional morbidity. Further studies are needed to determine the role of pelvic floor muscle function in motor performance in patients with ARM.

## Supplementary Information

**Additional file 1.** 

**Additional file 2.**

## Data Availability

All data generated or analysed during this study are included in this published article and its supplementary information files.

## Abbreviations

ARM: Anorectal malformation
HD: Hirschsprung’s disease
HrQOL: Health-related quality of life
PSK: Physisches Selbstkonzept (Physical self-concept)
PSK-K: Physisches Selbstkonzept-Kinder (Physical self-concept for children)
VACTERL: Acronym for (vertebral, anorectal, cardial, tracheoesophageal, limb) of associated malformations
95%-CI: 95%-Confidence interval
